# Comprehensive analysis of genomic variation, pan-genome and biosynthetic potential of *Corynebacterium glutamicum* strains

**DOI:** 10.1371/journal.pone.0299588

**Published:** 2024-05-08

**Authors:** Md. Shahedur Rahman, Md. Ebrahim Khalil Shimul, Md. Anowar Khasru Parvez

**Affiliations:** 1 Department of Genetic Engineering and Biotechnology, Jashore University of Science and Technology, Jashore, Bangladesh; 2 Department of Genetic Engineering and Biotechnology, Bioinformatics and Microbial Biotechnology Laboratory, Jashore University of Science and Technology, Jashore, Bangladesh; 3 Department of Microbiology, Jahangirnagar University, Savar, Dhaka, Bangladesh; Cardiff’s Metropolitan University: Cardiff Metropolitan University, UNITED KINGDOM

## Abstract

*Corynebacterium glutamicum* is a non-pathogenic species of the *Corynebacteriaceae* family. It has been broadly used in industrial biotechnology for the production of valuable products. Though it is widely accepted at the industrial level, knowledge about the genomic diversity of the strains is limited. Here, we investigated the comparative genomic features of the strains and pan-genomic characteristics. We also observed phylogenetic relationships among the strains based on average nucleotide identity (ANI). We found diversity between strains at the genomic and pan-genomic levels. Less than one-third of the *C*. *glutamicum* pan-genome consists of core genes and soft-core genes. Whereas, a large number of strain-specific genes covered about half of the total pan-genome. Besides, *C*. *glutamicum* pan-genome is open and expanding, which indicates the possible addition of new gene families to the pan-genome. We also investigated the distribution of biosynthetic gene clusters (BGCs) among the strains. We discovered slight variations of BGCs at the strain level. Several BGCs with the potential to express novel bioactive secondary metabolites have been identified. Therefore, by utilizing the characteristic advantages of *C*. *glutamicum*, different strains can be potential applicants for natural drug discovery.

## 1. Introduction

*Corynebacterium glutamicum* is a gram-positive, non-sporulating, non-pathogenic, and generally recognized as safe (GRAS) organism. It remains very robust against oxygen and substrate supply oscillation in the case of large-scale fermentations [1,2]. It is one of the most used microorganisms in industrial fermentation for producing amino acids, like lysine and glutamate, for decades [3,4]. *C*. *glutamicum* has undergone substantial modification to provide a wide range of beneficial products including chemicals, proteins, polymers, natural products, and biofuels [5–8]. Many studies of *C*. *glutamicum* have been published in the past decade [9], yet the genetic variations among the strains are unexplored.

Whole genomes of closely related and geographically co-occurring microbial strains show enormous variation within species, resulting from allelic and gene content changes [10–13]. However, it is challenging to distinguish between two lineages that are thought to be the same species yet have significantly different gene contents using conventional taxonomic approaches [14–16]. Hence, a better understanding of the genomic characteristics of different *C*. *glutamicum* strains is required.

Genes for the production, control, and resistance of secondary metabolites are often grouped to create biosynthetic gene clusters (BGCs) in microbial genomes [17]. Utilization of bioinformatics tools for the analysis of microbial genome sequences reported that a single genome may include 20–80 distinct BGCs [18]. On the other hand, a microorganism may possess certain BGCs but it may not express them in laboratory conditions [19,20]. Research in this area will support wet lab methods development for natural product (NPs) producing strains that have greater potential to produce new compounds [18]. In 2017, Yang and Yang conducted a comparative analysis of *C*. *glutamicum* genomes, providing insights into the genetic diversity and evolutionary relationships within this significant industrial bacterium [21]. The research also pinpointed crucial mutations associated with amino acid production in various genetically engineered strains. However, certain limitations and challenges persist. Specifically, the pan-genome analysis was conducted with a relatively limited number of strains, potentially not encompassing the entire spectrum of species diversity. Furthermore, the identification of BGCs remains incomplete, highlighting areas for further investigation. So, it should be helpful to use functional genomic approaches to identify those unidentified BGCs at the genomic level. Therefore, the BGCs distribution and evolutionary connections among the *C*. *glutamicum* strains need to be explored. The primary aim of this study is to analyse pan-genomic variations within different strains and explore the distribution patterns of BGCs.

## 2. Materials and methods

### 2.1 Whole genome comparison

Genomic datasets of *C*. *glutamicum* strains were collected from National Center for Biotechnology Information (NCBI) database (https://www.ncbi.nlm.nih.gov/datasets, accessed on 30^th^ May 2022). Initially, 65 complete genome sequences of *C*. *glutamicum* strains were retrieved in addition to the reference genome (In the NCBI database, *C*. *glutamicum* SCgG2 serves as the primary reference genome), all in FASTA format. The complete whole genome sequences of *C*. *glutamicum* were selected using the NCBI genome filter tool, and the assembly level was set to "complete". The choice of genomes was guided by contemporary research, emphasizing the pivotal role that high-quality genomes play in pangenome and genome mining analyses [22]. Consequently, this study excluded draft and scaffold level assemblies to ensure the integrity and reliability of the genomic data under examination. The use of complete genomes enhances the reliability and comprehensiveness of the study’s findings, contributing to a more accurate understanding of the *C*. *glutamicum*’s genetic diversity, functional capabilities, and evolutionary insights. Then, whole genome comparisons were executed using OrthoANI v0.5.0 with default parameters, which uses an enhanced pairwise average nucleotide identity (ANI) algorithm [23]. After the comparison, we selected 30 complete genomes, other 35 were discarded due to 100% similarity match. The program was also used to clear species boundaries and to get diversity at the genetic level among whole genomes (Table 1). In this way, redundancy was avoided and the genetic diversity of *C*. *glutamicum* was ensured.

**Table 1:** List of *C*. *glutamicum* strains used in this study with their metadata.

Accession no.	Strains	Source	Location	Reference
CP012194.1	CP	air	China	[24]
CP018175.1	XV	soil	China	[25]
AP017557.2	AJ1511		Japan	[26]
AP022856.1	ATCC 21799		Japan	[27]
CP004062.1	ZL-6	soil	China	
CP010451.1	B253		China	[28]
CP053188.1	BE		South Korea	
CP022614.1	ATCC 14067	rotten onion	Guangzhou, Guangdong, China	
CP014984.1	YI	soil	China	[25]
NC_009342.1	R			[29]
CP004047.1	SCgG1	soil	China	
CP004048.1	SCgG2	soil	China	
CP013991.1	USDA-ARS-USMARC-56828	nasopharynx of calf	Tennessee, USA	
NZ_CP073911.1	CGMCC1.15647		Jiangsu, China	[30]
CP068290.1	ATCC 21573			
CP012297.1	B414	soil		
CP012298.1	CICC10064	soil		
CP005959.1	MB001			[31]
CP020658.1	TQ2223	soil	Tianjin, China	
CP025533.1	ATCC 13032		South Korea	[32]
CP025534.1	HA		South Korea	[32]
NZ_CP059382.1	BCA		Portugal	[33]
CP017995.1	C1	engineered derivative of C. glutamicum ATCC 13032	Germany	[34]
CP080542.1	CR101	lab strain	Bielefeld University, Germany	[35]
CP041729.1	JH41		Daejeon, South Korea	[36]
CP007724.1	AR1		Daejeon, South Korea	[37]
CP007722.1	ATCC 21831			[37]
CP020033.1	TCCC11822	soil	Tianjin, China	
CP016335.1	ATCC 13869	soil	Japan	[38]
NZ_CP022394.1	WM001	soil	Wuxi, Jiangsu, China	[39]

### 2.2 Genome annotation

The process of locating and designating all the pertinent features on a genomic sequence is known as genome annotation [40]. Selected whole genome sequences were re-annotated using Prokka v1.14.6 with default parameters [41]. Prokka uses BLAST+ and identifies best match of annotated protein and candidate genes from various databases [41]. Prokka and FragGeneScan v1.31 were used with default parameters to identify the number of genes in each genome [42]. It uses a novel gene prediction technique and improved prediction of the protein-coding region in short reads by combining codon usages and sequencing error models in a Hidden Markov Model (HMM) [42].

### 2.3 Pan-genome analysis

Pan-genomic analysis was conducted utilizing Roary v3.11.2 (with default parameters), a robust computational tool specifically designed for such analyses. Roary classifies genes into distinct categories, including ’core genes’, ’cloud genes’, ’shell genes’, and ’soft-core genes’, employing a rigorous computational framework [43]. Bacterial Pan-genome Analysis tool (BPGA v1.3) [44] was employed for the systematic classification of orthologous genes into core, accessory, and unique genomes. Subsequently, strains containing a relatively higher number of unique genes were subjected to annotation using the blast algorithm against the Clusters of Orthologous Genes (COG) database [45]. To gain in-depth insights into the functional aspects of these genes, further analyses were conducted utilizing the blast algorithm against both the COG and Kyoto Encyclopedia of Genes and Genomes (KEGG) database [46]. The estimation of the pan-genome and core genome was performed using the USEARCH v11.0.667 [47] program available in BPGA, employing a 50% sequence identity cut-off. The resulting data were then subjected to nonlinear fitting based on the model extrapolation of the pan-genome and core genome, ensuring a robust and comprehensive analysis of the bacterial genomic elements under investigation [44,48].

### 2.4 Phylogeny

FastTree v2.1.11 (with default parameters) was used to generate phylogenetic tree, which uses the maximum-likelihood method with generalized time-reversible (GTR) models of nucleotide evolution [49]. iTOL, an online platform was used to visualize the phylogenetic tree [50].

### 2.5 Identification of BGCs

We used three platforms to predict BGCs, which can accurately predict microbial secondary metabolite encoding regions by using sophisticated computer model services [51]. These are namely antiSMASH 6 (https://antismash.secondarymetabolites.org/, accessed on 9^th^, June 2022) [52], PRISM 4 (http://prism.adapsyn.com, accessed, accessed on 28^th^, June 2022) [53] and BAGEL4 (http://bagel4.molgenrug.nl, accessed on 29^th^, June 2022) [54]. BGC boundaries in this study was detected using antiSMASH 6, a computational tool that employs several techniques. Firstly, antiSMASH determines BGC boundaries based on the physical distance to core domains within the analyzed sequences [55]. It utilizes ClusterCompare output, conducting a search of all gene products against a database comprising highly conserved enzyme Hidden Markov Model (HMM) profiles indicative of specific BGC types [56]. The tool applies pre-defined cluster rules to identify individual protoclusters encoded in the genomic region. To standardize gene locations, antiSMASH employs a reference genome as a common coordinate system, allowing for the normalization of gene positions. Additionally, antiSMASH maps genomes of other strains containing the same or similar BGCs to the reference genome through alignment tools. This enables the identification and comparison of genomic regions corresponding to the BGCs across different strains in relation to the reference genome [52]. PRISM 4 predicts BGCs by analysing open reading frames from various databases [53]. BAGEL4 identifies ribosomally synthesized and post-translationally modified peptides (RiPPs), and Bacteriocin. It discovers gene clusters by using peptide database and/or through HMM motifs that are present in relevant contextual genes, augmented with literature references and links to UniProt and NCBI [54].

### 2.6 Genomic analysis and single nucleotide polymorphism identification

Genome comparisons among *C*. *glutamicum* strains were conducted using BLAST Ring Image Generator (BRIG-0.95-dist) with default settings. BRIG plays a pivotal role in facilitating the assessment of genotypic distinctions within closely related prokaryotic organisms [57]. In this study, we utilized the Mauve genome alignment system to analyze *C*. *glutamicum* strains [58]. Throughout evolution, microbial genomes can experience substantial mutations, including rearrangements and lateral transfers, leading to notable differences in gene order and content among closely related organisms. Mauve, a powerful tool, was employed to identify these events, enabling comprehensive comparisons of multiple microbial genomes, even in the presence of high recombination rates. The Mauve system was configured with default settings, employing deed weight, full alignment, and iterative refinement techniques.

In our study, we utilized single nucleotide polymorphism (SNP) analysis as a methodology to discern genetic variations within the strains of *C*. *glutamicum*. The identification of variants among these strains was conducted through the implementation of Snippy v4.6.0 [59], with the reference sequence being *C*. *glutamicum* SCgG2. Notably, the prediction of Core SNPs was an additional aspect addressed in our analysis, employing the same Snippy tool for this specific task. This comprehensive approach allowed for a detailed exploration of genetic diversity and core variations within the *C*. *glutamicum* strains under investigation.

### 2.7 Identification of horizontal gene transfer

The prediction of horizontally transferred genes was carried out using HGTector v2.0b3 (with default settings) [60]. The analysis focused on identifying horizontal gene transfer (HGT) events within *C*. *glutamicum* AJ1511 and *C*. *glutamicum* AR1 genomes. A search was conducted utilizing the default remote database with stringent criteria, requiring a minimum identity and coverage of greater than 50%. The analysis was executed with default parameters to ensure comprehensive and accurate detection of potential HGT events in the studied strains.

### 2.8 Pathogenic and non-pathogenic properties and plasmid typing

The prediction of pathogenicity for the chosen strains was carried out using the PathogenFinder web tool [61]. This tool employs a predictive model that considers both the probability score and the resemblance to known pathogenic species in order to assess the likelihood of pathogenicity.

The plasmid sequences were obtained from the NCBI database. To ascertain the classification of plasmids, Plasmid Multi-Locus Sequence Typing (Plasmid MLST) was employed. Plasmid MLST is a molecular typing method that analyzes specific genetic markers across plasmids, providing insights into their type and lineage. This approach aids in categorizing plasmids based on their sequence diversity and assists in understanding the genetic variation and relationships among different plasmid strains.

## 3. Results

Demographic information about the strains used in this study are listed in **Table 1**. Among the strains, 11 were isolated from soil and others were isolated from air, mucus, rotten onion and lab strains. Among them 20 strains were isolated from Asian countries, 2 strains were isolated from Germany, and 1 strain from Portugal and United States of America each. Others origin are unknown.

### 3.1 Whole genome comparison

**Fig 1:**
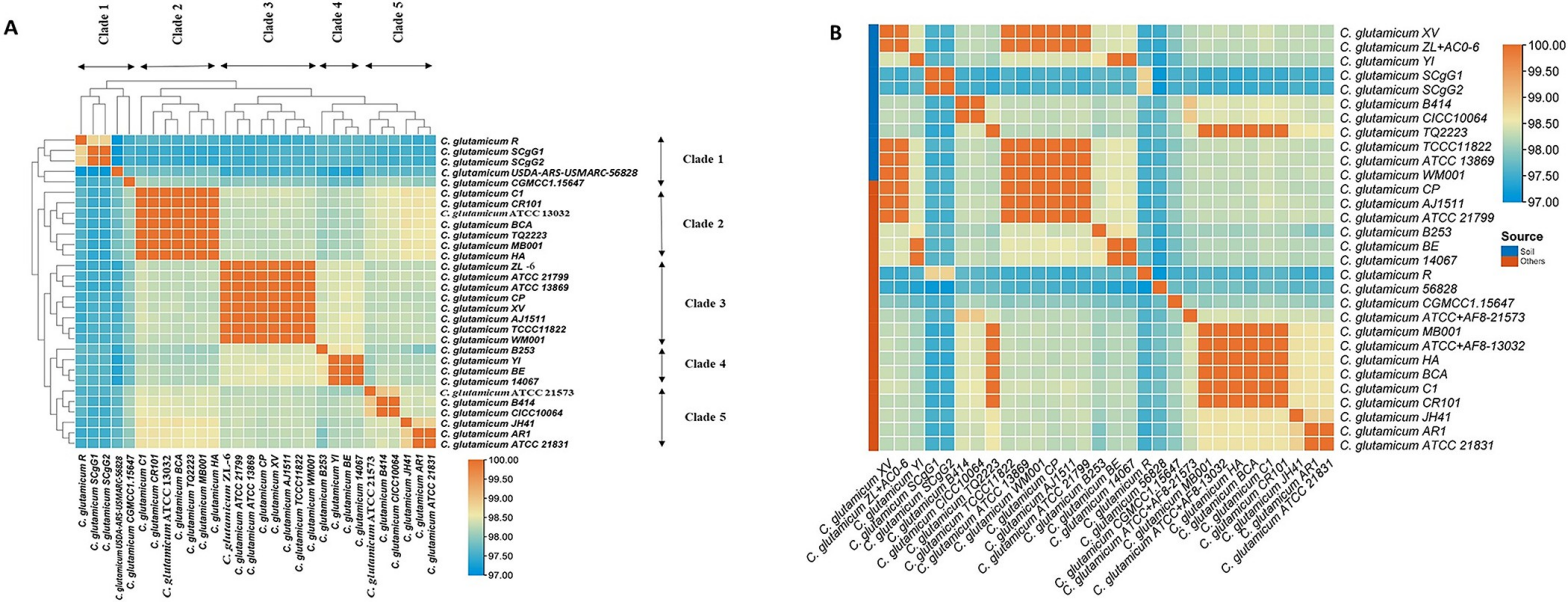
(A) ANI based whole genome comparison of *C*. *glutamicum* strains. The linkage method was average linkage, which calculates the average distance between all pairs of points in two clusters. The distance metric was Euclidean distance, which measures the straight-line distance between two points in a multidimensional space. The distance threshold was 0.5, which means that clusters with a distance less than or equal to 0.5 were merged together. This resulted in five clades, as shown by the horizontal dashed line in the plot. (B) ANI comparisons conducted among strains isolated from soil environments and strains isolated from both soil and non-soil environments within *C*. *glutamicum*.

The degree of relatedness in the studied strains were identified by calculating ANI. ANI also clarifies whether the genomes reside in the same species by a cut-off values of ≥ 95% for same species. Our studied genomes have shown higher than 97% ANI values, confirming that all the genomes of the strains belong to the same species (**S1 Table**). A heat-map generated from the ANI scores have shown (**Fig 1**). There are five sub-groups in the heat-map and can be called as five clades. The clades were extracted from pairwise ANIs by using a hierarchical clustering algorithm with a cut-off value of 0.5. This means that strains with ANI values higher than 0.5 were grouped together in the same clade. The clades do not seem to have a strong correlation with the source and geographic location of the genomes. For example, clade 1 contains strains from soil and mucus sources, and from China and USA locations. Clade 2 contains strains from soil sources, and from Germany, China, South Korea, and Portugal locations. Clade 3 contains strains from soil, air and lab sources, and from China and Japan locations. Clade 4 contains strains from soil and rotten onion sources, and from China and South Korea locations. Clade 5 contains strains from soil sources, and from South Korea locations. Strains belonging to clade 1 (R, SCgG1, SCgG2) exhibit big genome size, a notable presence of multiple copies of NAPAA biosynthetic gene clusters (BGCs) and concurrently possess betalactone BGCs. This characteristic occurrence may contribute to their distinctiveness as outliers within the broader spectrum of analyzed genomes.

Fig 1B represents the ANI comparisons among various *C*. *glutamicum* strains isolated from different sources, including soil and non-soil environments. The ANI values revealed significant insights into the genetic relationships among these strains, shedding light on the impact of isolation sources on their genetic similarity. When we performed a more detailed ANI analysis, we observed that the strains isolated from soil environments, such as *C*. *glutamicum* XV, *C*. *glutamicum* ZL, and *C*. *glutamicum* YI, exhibited ANI values close to 98%, indicating a high genetic similarity. This suggests a common genetic background among these soil-isolated strains. On the other hand, when comparing soil-isolated strains with those from non-soil sources, the ANI values were notably lower, hovering around 97%. This discrepancy underscores the genetic divergence between strains from soil and non-soil origins. Such divergence could potentially be attributed to environmental factors and selective pressures specific to these habitats, leading to genetic adaptations unique to each niche.

### 3.2 Comparative genomic features of *C*. *glutamicum* strains

**Fig 2:**
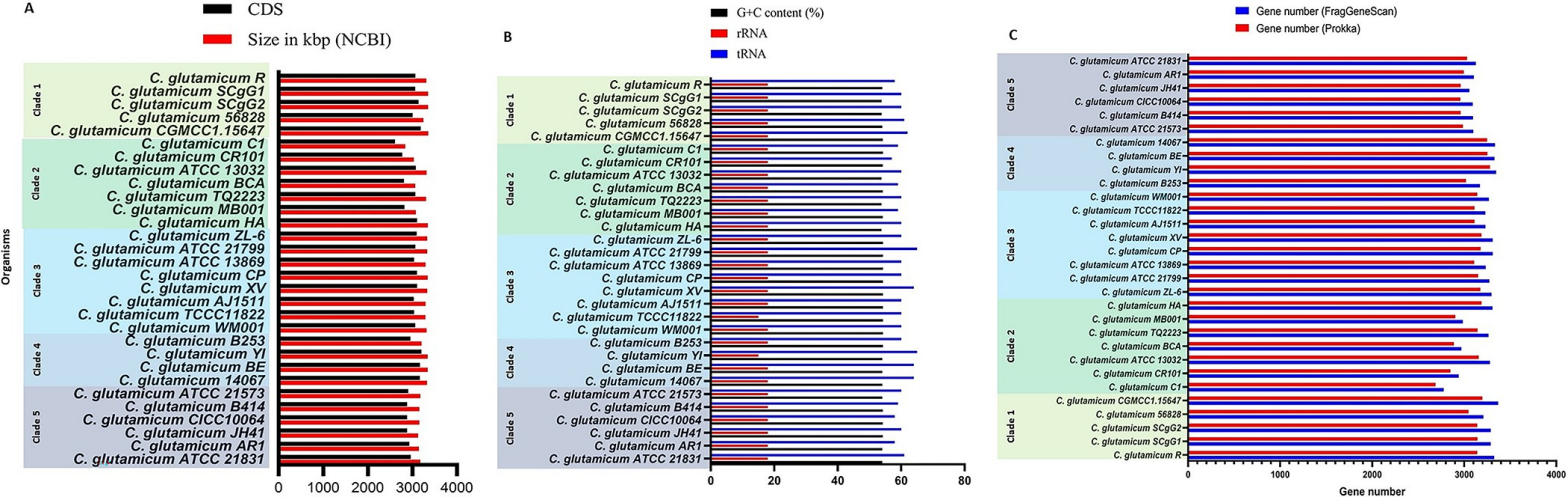
Overview of genomic features. (A) Genome size and CDS. (B) Genomic features (tRNA, rRNA and GC content (%)). (C) Gene number.

The average genome size of *C*. *glutamicum* strains was 3.24 Mbp (ranging from 2.84 Mbp to 3.36 Mbp) (**Fig 2A and S2 Table**). Coding sequence (CDS) count was predicted with the highest 3200 CDS to lowest 2610 CDS with a mean of 3007 CDS among the whole genomes (**Fig 2A and S2 Table**). The average GC content was 54.15% among the genomes, and the approximate number of tRNA genes ranged from 57 to 65, while the predicted rRNA genes were 18 among all 28 strains excluding strain TCCC11822 and strain YI having 15 rRNA genes (**Fig 2B and S2 Table**).

The gene count, as determined by Prokka, displayed a range of 2688 to 3281 genes, with a mean value of 3078 genes per genome. In contrast, gene predictions through FragGeneScan exhibited a range of 2778 to 3369 genes, yielding a mean of 3197 genes per genome. It is noteworthy that Prokka’s predictions resulted in a comparatively lower gene count than those obtained via FragGeneScan **(Fig 2C and S2 Table**).

### 3.3 Pan-genome analysis

**Fig 3:**
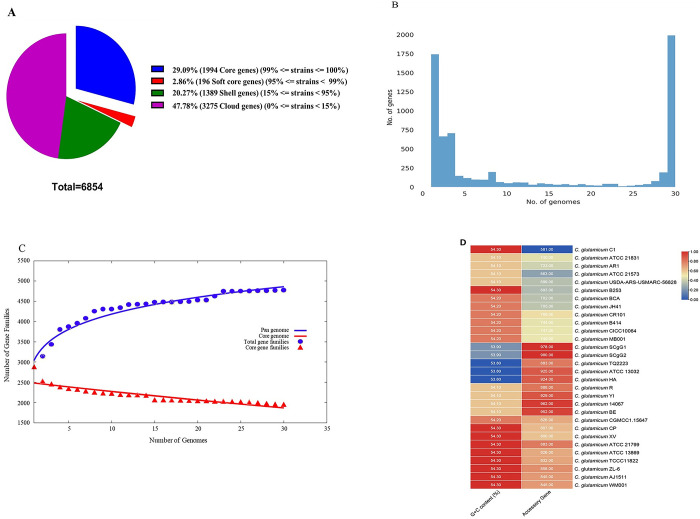
Pan-genome analysis of *C*. *glutamicum*. (A) The number of core genes, soft core genes, shell genes and cloud genes in the pan genome. (B) Gene frequency versus genomes number. (C) The pan genome profile trends obtained using BPGA v1.3. (D) Genomic G+C content (%) and accessory gene counts in various *C*. *glutamicum* strains.

Roary analysis predicted total 6854 protein-coding gene sequences. The number of core genes was 29% (99% < = strains < = 100%), the number of soft core genes was 2.86% (95% < = strains < 99%), the number of shell genes was 20.27% (15% < = strains < 95%), and the number of cloud genes was 47.78% (0% < = strains < 15%) (**Fig 3A**).

**Fig 4:**
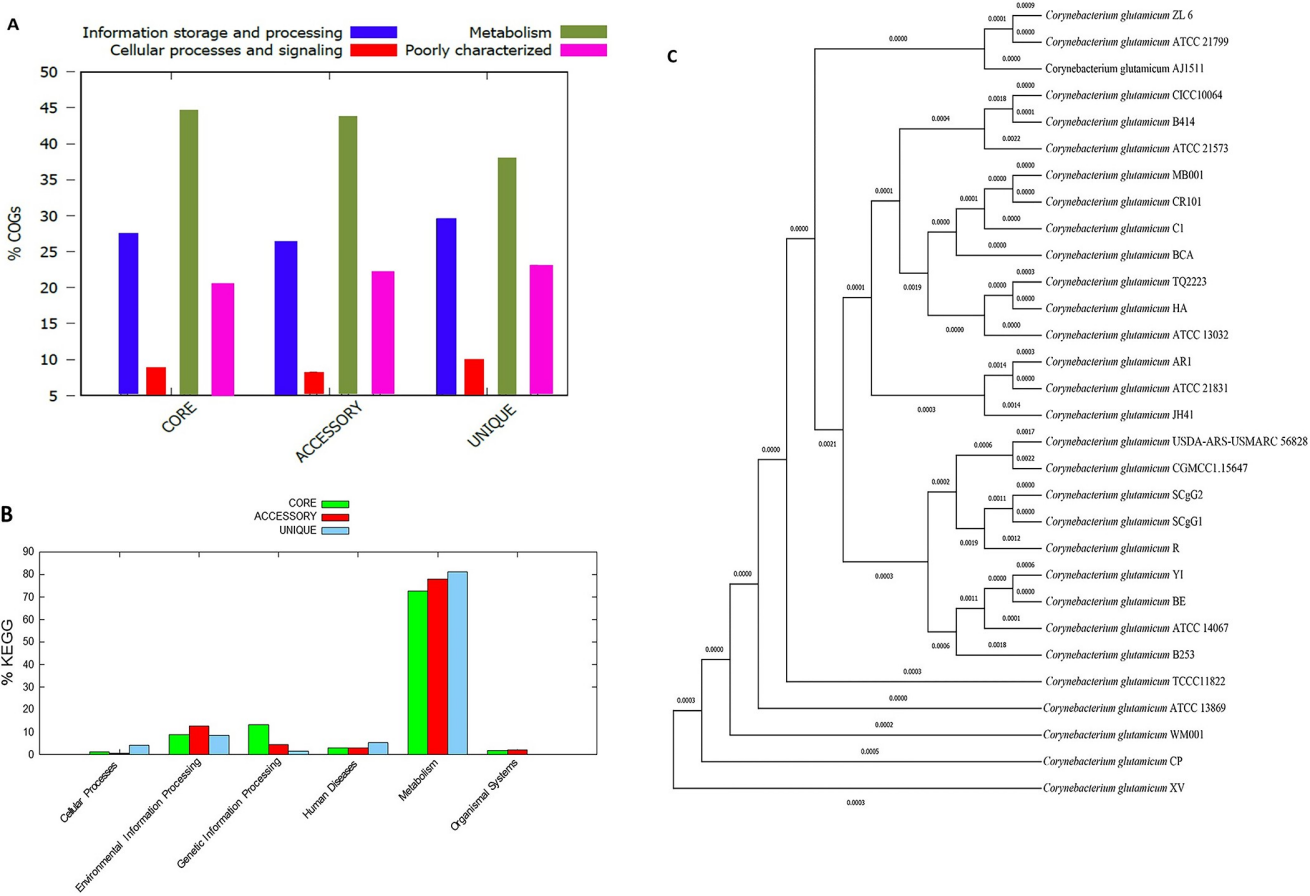
(A) COG distribution of core, accessory and unique genes. (B) KEGG distribution of core, accessory and unique genes. (C) Phylogenetic analysis of *C*. *glutamicum* strains based on core genes.

The high number of cloud genes exhibit significant variation and shows the ’open’ nature of the *C*. *glutamicum* pan-genome (**Fig 3B**). The pan-genome of *C*. *glutamicum* was analysed using an empirical power law regression function based on the Allometric1 model (f(x) = 3059.17x^0.136303^). The obtained parameter exponent (0.136303), falling between 0 and 1 and indicates that the pan-genome grows more slowly than other bacteria (possibly due to slower genetic diversification), but will grow indefinitely nonetheless (**Fig 3C**). In the context of Heaps’ law, an ’open’ pan-genome suggests the presence of a substantial and indeterminate number of additional genes, with its size potentially increasing boundlessly as more strains are included in the analysis [62–64]. *C*. *glutamicum* strains TQ2223, ATCC 13032, and HA exhibit a relatively low GC content coupled with a notable abundance of accessory genes (883, 925, and 924, respectively). Among these strains, *C*. *glutamicum* SCgG2 displays the lowest GC content and concurrently possesses the highest number of accessory genes (**Fig 3D**). In Fig 4A and 4B, the distribution of COG and KEGG categories for core, accessory, and unique genes is illustrated. Fig 4C displays the phylogenetic relationships among *C*. *glutamicum* strains based on core genes.

The core genome is primarily associated with essential biological functions such as amino acid transport and metabolism, translation, ribosomal structure and biogenesis, transcription, carbohydrate transport and metabolism, inorganic ion transport and metabolism, and post-translational modification, protein turnover, and chaperones. Simultaneously, the number of unique genes within *C*. *glutamicum* genomes varied significantly, indicating individual differences and a relatively high level of genomic diversity. This variability suggests their potential adaptation to diverse and extreme environments. Furthermore, KEGG pathway analysis revealed that these unique genes are involved in various biological processes related to metabolism, environmental information processing, and cellular processes.

### 3.4 Diversity and abundance of potential BGCs

**Fig 5:**
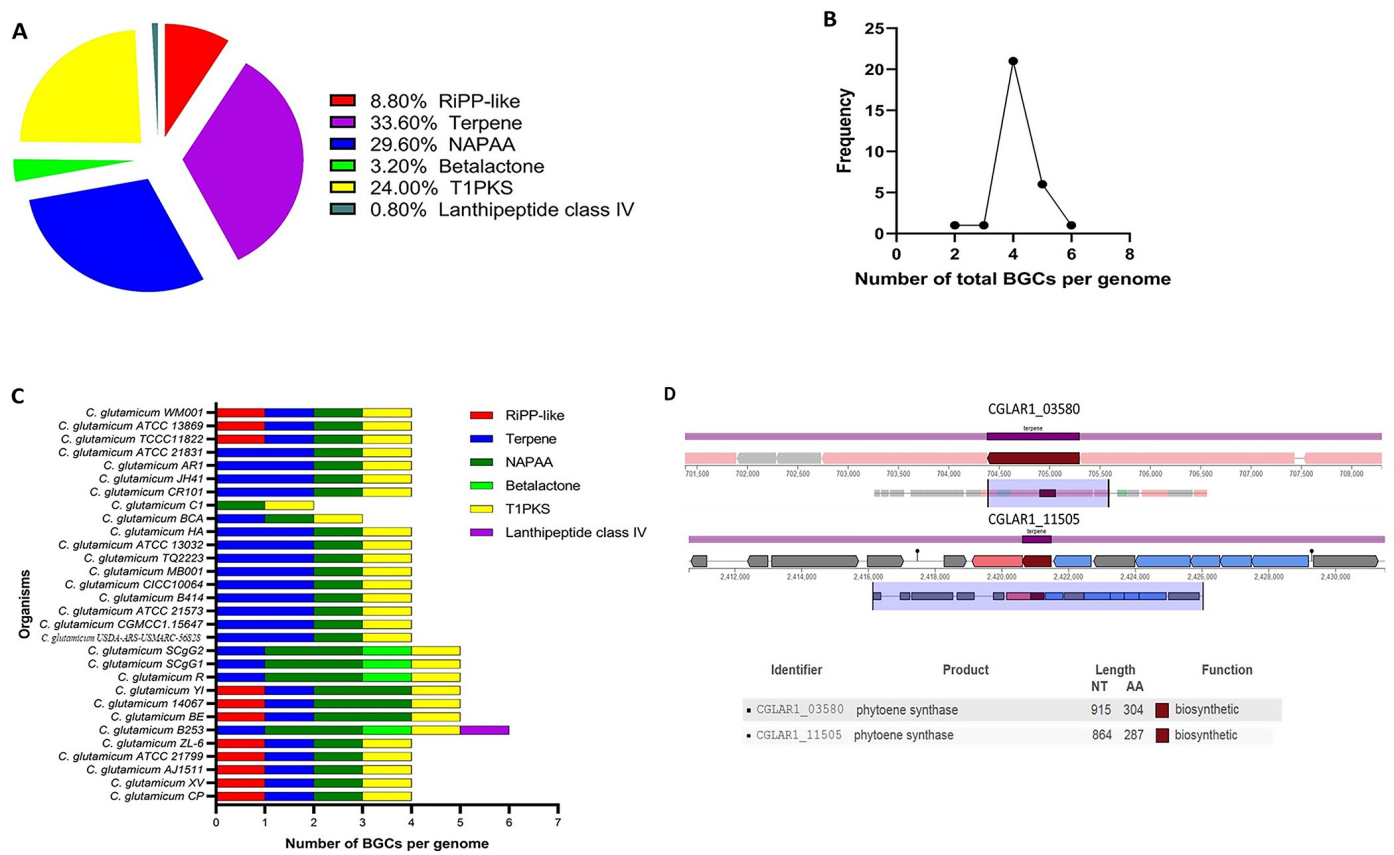
BGCs among *C*. *glutamicum* strains. (A) Distribution of different classes of BGCs among *C*. *glutamicum* strains. (B) BGCs frequency per genome. (C) Different classes of BGCs occurrence in the genomes. (D) BGCs of *C*. *glutamicum* AR1.

AntiSMASH prediction identified six different classes of BGCs among the whole genomes. Identified BGCs include terpene, non-alpha poly-amino acids like e-polylysin (NAPAA), Betalactone, type 1 polyketide synthase (T1PKS), other unspecified ribosomally synthesized and post-translationally modified peptide product cluster (RiPP-like), and lanthipeptide class IV. Terpene synthesis BGCs were the most abundant BGCs in the genomes. NAPAA and T1PKS were the second most abundant BGCs, and collectively these 3 BGCS (Terpene, NAPAA, and T1PKS) comprised over 87% of the BGCs among the 30 strains of *C*. *glutamicum* (**Fig 5 and S3 Table**).

The strains harbour 2 to 6 BGCs and maximum 21 strains harbour 4 BGCs. The highest 6 BGCs were found in strain B253 and the lowest 2 BGCs in strain C1. Besides, 6 strains which are strain BE, 14067, YI, R, SCgG1, and SCgG2 contain 5 BGCs in their genomes (**Fig 5B and 5C**). Betalactone and lanthipeptide class IV BGCs were the most common BGCs. Betalactone BGCs were predicted in strain R, B253, SCgG1, and SCgG2, while lanthipeptide class IV BGCs were only found in strain B253 (**Fig 5C and S4 Table**). *C*. *glutamicum* strains have also been found harbouring double copies of same BGCs class in 20 strains. Terpene class BGCs were observed to be duplicated in up to 13 strains, exemplified by strain AR1, which possesses two distinct terpene class BGCs (identified as CGLAR1_11505 and CGLAR1_03580). Both clusters encode phytoene synthase, yet their gene products differ in size, with lengths of 287 and 304 amino acids, respectively (**Fig 5D**). In contrast, NAPAA class BGCs were identified as duplicated entities in 7 strains, as illustrated in Fig 5C and detailed in S4 Table. The antiSMASH analysis of *C*. *glutamicum* strains identified terpene BGCs across different clades, demonstrating considerable diversity in encoded compounds. In Clade 1, strains like CGMCC1.15647, USDA-ARS-USMARC-56828, R, SCgG2, and SCgG1 produced phytoene/squalene synthase. CGMCC1.15647 exhibited multiple copies of phytoene/squalene synthase genes in different genome regions, indicating intra-strain variability. Clade 2 strains, including ATCC_13032, HA, TQ2223, MB001, CR101, and BCA, showed varying similarity scores for phytoene synthase, highlighting potential enzyme differences. Clade 3 strains, except C1, produced phytoene synthase, with variations seen in strains JH41 and B414. ATCC_21573 produced phytoene/squalene synthase. These findings suggest nuanced terpene production within clades, with variability in gene length and location across strains, underscoring the intricate diversity in *C*. *glutamicum* terpene biosynthesis (**Table 2**).

**Table 2:** Predicted terpene BGCs in different clades of *C*. *glutamicum* strains.

No of Clade	Name of Organism	Region	Location		Length		Compound			Similarity
			Start	End	NT	AA	Most Similar	Type	Product	
1	*C*. *glutamicum* CGMCC1.15647	Region 1	717,181	736,285	918	305	Carotenoid	Terpene	phytoene/squalene synthase family protein	100%
		Region 2	2,558,650	2,569,225	576	191	Carotenoid	Terpene	phytoene/squalene synthase family protein	-
			2,569,222	2,569,473	252	83	Carotenoid	Terpene	phytoene/squalene synthase family protein	-
	*C*. *glutamicum* USDA-ARS-USMARC-56828	Region 1	773,090	774,004	915	304	Carotenoid	Terpene	phytoene synthase	100%
		Region 2	2,488,448	2,489,271	824	274	Carotenoid	Terpene	phytoene synthase	25%
	*C*. *glutamicum* R	Region 2	816,127	817,041	915	304	Carotenoid	Terpene	phytoene/squalene synthase family protein	100%
	*C*. *glutamicum* SCgG2	Region 2	828,029	828,946	918	305	Carotenoid	Terpene	phytoene synthase	100%
	*C*. *glutamicum* SCgG1	Region 2	828,028	828,945	918	305	Carotenoid	Terpene	phytoene synthase	100%
2	*C*. *glutamicum* ATCC_13032	Region 1	636,907	637,821	915	304	Carotenoid	Terpene	phytoene/squalene synthase family protein	100%
		Region 2	2,581,072	2,581,980	909	302	Carotenoid	Terpene	phytoene/squalene synthase family protein	25%
	*C*. *glutamicum* HA	Region 1	45,599	46,507	909	302	Carotenoid	Terpene	phytoene/squalene synthase family protein	25%
		Region 4	1,416,327	1,417,241	915	304	Carotenoid	Terpene	phytoene/squalene synthase family protein	100%
	*C*. *glutamicum* TQ2223	Region 1	189,322	190,230	909	302	Carotenoid	Terpene	phytoene synthase	25%
		Region 2	2,098,009	2,098,923	915	304	Carotenoid	Terpene	phytoene synthase	100%
	*C*. *glutamicum* MB001	Region 1	638,057	638,971	915	304	Carotenoid	Terpene	phytoene synthase	100%
		Region 2	2,342,054	2,342,917	864	287	Carotenoid	Terpene	geranylgeranyl- diphosphategeranylgeranyltransferase	25%
	*C*. *glutamicum* C1	-	-	-	-	-	-	-	-	-
	*C*. *glutamicum* CR101	Region 1	631,448	632,362	915	304	Carotenoid	Terpene	phytoene synthase	100%
		Region 2	2,315,455	2,316,318	864	287	Carotenoid	Terpene	geranylgeranyl- diphosphategeranylgeranyltransferase	25%
	*C*. *glutamicum* BCA	Region 1	2,325,423	2,326,286	864	287	Carotenoid	Terpene	phytoene/squalene synthase family protein	25%
3	*C*. *glutamicum* XV	Region 2	749,608	750,525	918	305	Carotenoid	Terpene	phytoene synthase	100%
	*C*. *glutamicum* CP	Region 2	785,574	786,491	918	305	Carotenoid	Terpene	phytoene synthase	100%
	*C*. *glutamicum* WM001	Region 1	295,442	296,359	918	305	Carotenoid	Terpene	phytoene/squalene synthase family protein	100%
	*C*. *glutamicum* ATCC_13869	Region 2	744,821	745,738	918	305	Carotenoid	Terpene	phytoene synthase	100%
	*C*. *glutamicum* AJ1511	Region 1	746,437	747,354	918	305	Carotenoid	Terpene	phytoene synthase	100%
	*C*. *glutamicum* ZL-6	Region 2	753,776	754,693	918	305	Carotenoid	Terpene	phytoene synthase	100%
	*C*. *glutamicum* ATCC_21799	Region 4	2,581,219	2,582,136	918	305	Carotenoid	Terpene	phytoene synthase	87%
	*C*. *glutamicum* TCCC11822	Region 2	748,242	749,159	918	305	Carotenoid	Terpene	phytoene synthase	100%
4	*C*. *glutamicum* B253	Region 1	697,068	697,985	918	305	Carotenoid	Terpene	phytoene synthase	100%
	*C*. *glutamicum* BE	Region 1	294,621	295,538	918	305	Carotenoid	Terpene	phytoene/squalene synthase family protein	100%
	*C*. *glutamicum* YI	Region 3	704,032	704,949	918	305	Carotenoid	Terpene	phytoene synthase	100%
	*C*. *glutamicum* ATCC_14067	Region 3	705,614	706,531	918	305	Carotenoid	Terpene	phytoene/squalene synthase family protein	100%
5	*C*. *glutamicum* AR1	Region 1	704,384	705,298	915	304	Carotenoid	Terpene	phytoene synthase	100%
		Region 2	2,420,624	2,421,487	864	287	Carotenoid	Terpene	phytoene synthase	25%
	*C*. *glutamicum* ATCC_21831	Region 1	735,201	736,115	915	304	Carotenoid	Terpene	phytoene synthase	100%
		Region 2	2,451,884	2,452,747	864	287	Carotenoid	Terpene	phytoene synthase	25%
	*C*. *glutamicum* JH41	Region 1	649,099	649,977	879	292	Carotenoid	Terpene	phytoene/squalene synthase family protein	25%
		Region 4	2,060,429	2,061,343	915	304	Carotenoid	Terpene	phytoene synthase	100%
	*C*. *glutamicum* B414	Region 1	723,465	724,379	915	304	Carotenoid	Terpene	phytoene synthase	100%
		Region 2	2,428,684	2,429,508	825	274	Carotenoid	Terpene	phytoene synthase	25%
	*C*. *glutamicum* CICC10064	Region 1	725,672	726,586	915	304	Carotenoid	Terpene	phytoene synthase	100%
		Region 2	2,431,451	2,432,275	825	274	Carotenoid	Terpene	phytoene synthase	25%
	*C*. *glutamicum* ATCC_21573	Region 1	697,484	698,398	915	304	Carotenoid	Terpene	phytoene/squalene synthase family protein	100%
		Region 2	2,450,615	2,451,478	864	287	Carotenoid	Terpene	phytoene/squalene synthase family protein	25%

Similarity: Similarity percentage with the reference database.

Region: Identified BGCs location in genome.

The analysis conducted using BAGEL4 showcased the antimicrobial capabilities within *C*. *glutamicum* strains. Clade 1 and clade 2 strains were devoid of identifiable specific bacteriocin BGCs. Clade 3 and clade 4 strains exhibited a shared putative bacteriocin BGC named "Lactococcin_972," indicating potential similar antimicrobial characteristics. Conversely, Clade 5, akin to clade 1 and 2, did not demonstrate distinct bacteriocin BGCs (**Table 3**).

**Table 3:** Predicted bacteriocin BGC in *C*. *glutamicum* strains.

No of Clade	Name of Organism	Gene Length		Compound	
		Start	End	Type	Name
1	*C*. *glutamicum* CGMCC1.15647	-	-	-	-
	*C*. *glutamicum* USDA-ARS-USMARC-56828	-	-	-	-
	*C*. *glutamicum* R	-	-	-	-
	*C*. *glutamicum* SCgG2	-	-	-	-
	*C*. *glutamicum* SCgG1	-	-	-	-
2	*C*. *glutamicum* ATCC_13032	-	-	-	-
	*C*. *glutamicum* HA	-	-	-	-
	*C*. *glutamicum* TQ2223	-	-	-	-
	*C*. *glutamicum* MB001	-	-	-	-
	*C*. *glutamicum* C1	-	-	-	-
	*C*. *glutamicum* CR101	-	-	-	-
	*C*. *glutamicum* BCA	-	-	-	-
3	*C*. *glutamicum* XV	244312	244611	putative_bacteriocin	Lactococcin_972
	*C*. *glutamicum* CP	245624	245923	putative_bacteriocin	Lactococcin_972
	*C*. *glutamicum* WM001	3092978	3093184	putative_bacteriocin	Lactococcin_972
	*C*. *glutamicum* ATCC_13869	244322	244621	putative_bacteriocin	Lactococcin_972
	*C*. *glutamicum* AJ1511	245938	246237	putative_bacteriocin	Lactococcin_972
	*C*. *glutamicum* ZL-6	245649	245948	putative_bacteriocin	Lactococcin_972
	*C*. *glutamicum* ATCC_21799	2077312	2077611	putative_bacteriocin	Lactococcin_972
	*C*. *glutamicum* TCCC11822	244425	244631	putative_bacteriocin	Lactococcin_972
4	*C*. *glutamicum* B253	206829	207086	putative_bacteriocin	Lactococcin_972
	*C*. *glutamicum* BE	3167629	3167835	putative_bacteriocin	Lactococcin_972
	*C*. *glutamicum* YI	235165	235464	putative_bacteriocin	Lactococcin_972
	*C*. *glutamicum* ATCC_14067	236772	237071	putative_bacteriocin	Lactococcin_972
5	*C*. *glutamicum* AR1	-	-	-	-
	*C*. *glutamicum* ATCC_21831	-	-	-	-
	*C*. *glutamicum* JH41	-	-	-	-
	*C*. *glutamicum* B414	-	-	-	-
	*C*. *glutamicum* CICC10064	-	-	-	-
	*C*. *glutamicum* ATCC_21573	-	-	-	-

**Fig 6:**
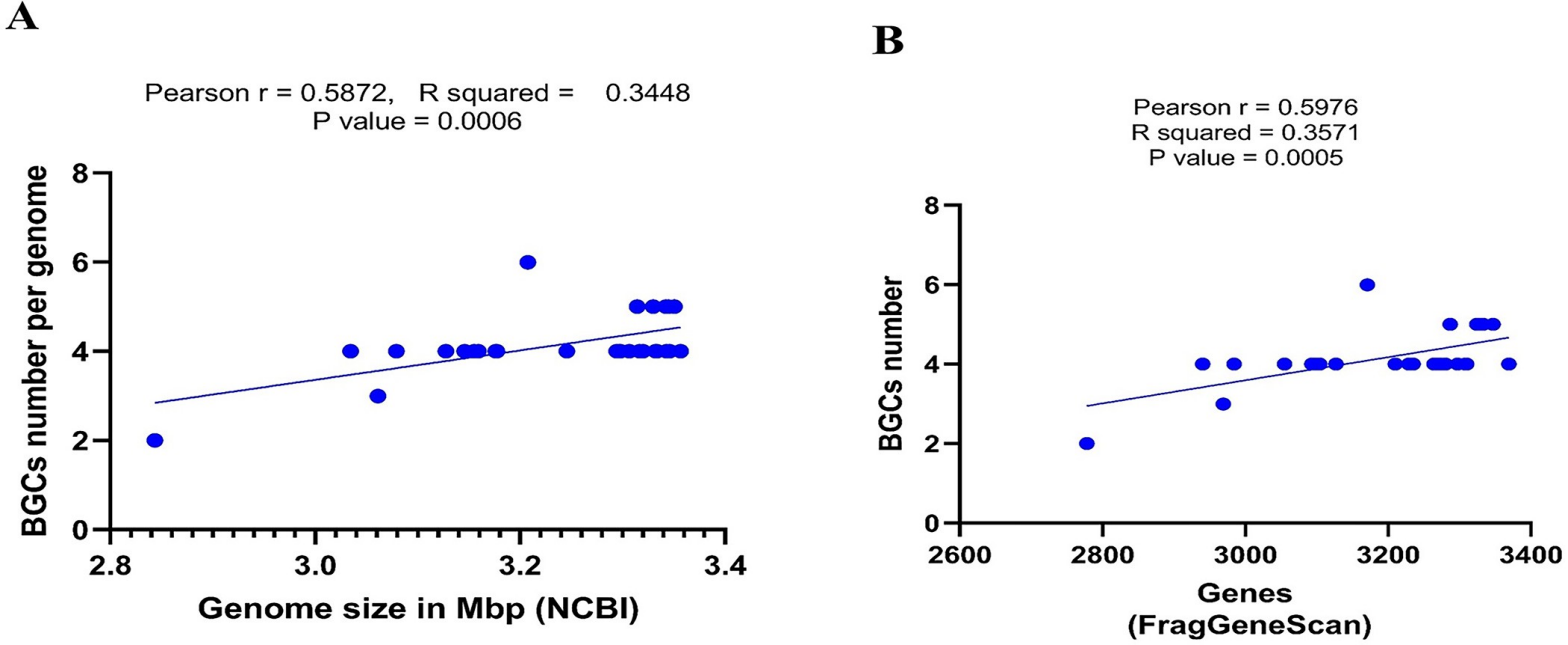
BGCs distribution in *C*. *glutamicum* strains. (A) Correlation of BGCs and genome size. (B) Correlation of BGCs and gene number.

**Fig 7:**
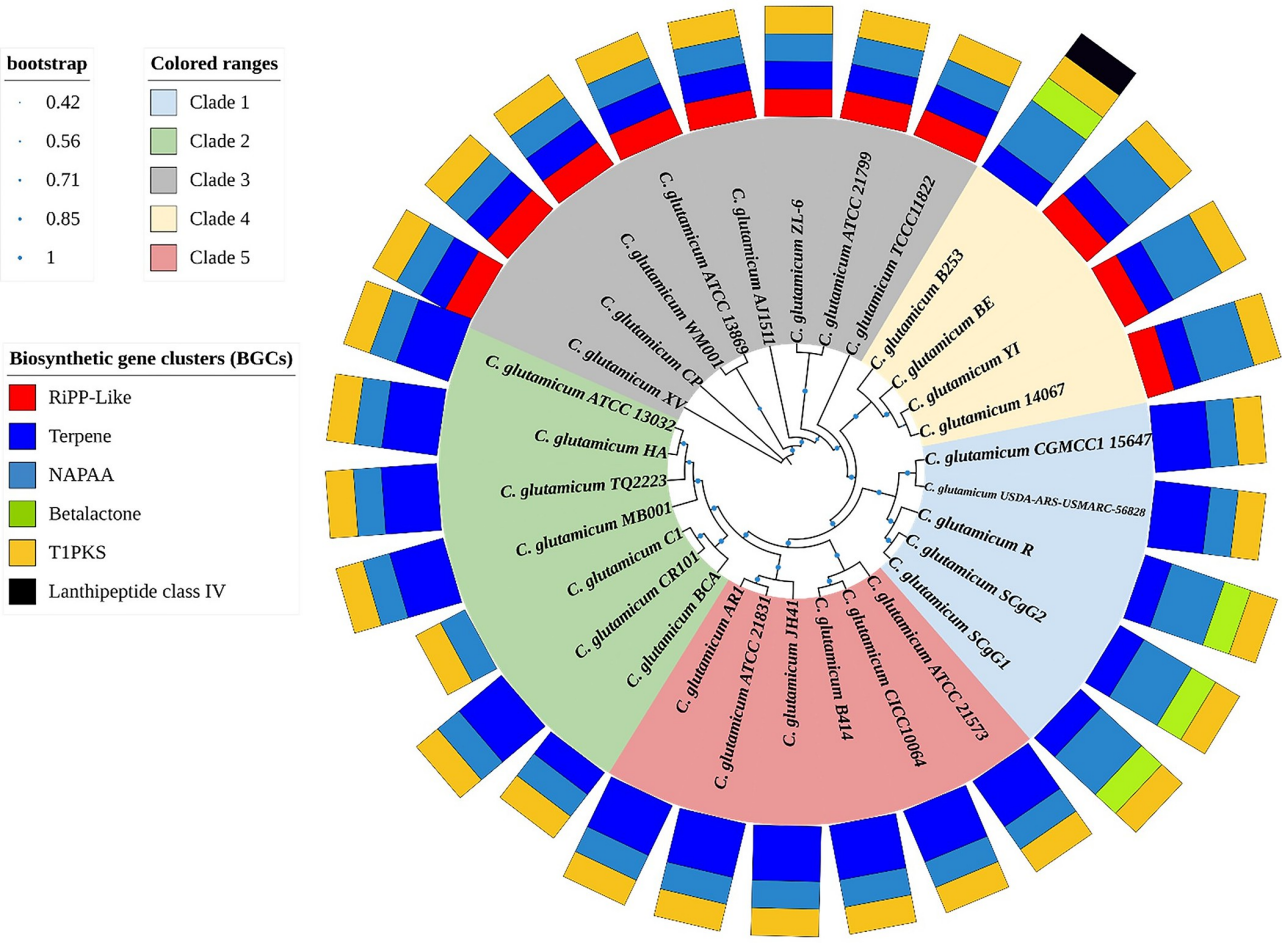
Major classes of BGCs in the genomes of *C*. *glutamicum* strains with phylogenetic distribution. These BGC classes are categorized into five clades, each delineated based on their specific biosynthetic gene content.

Correlation between BGCs number with genome size and total gene count indicates a moderate positive correlation (R^2^ = 0.349 and R^2^ = 0.358 respectively) (**Fig 6**). The diversity of BGCs among the strains with phylogenetic relationship were shown in five clades (**Fig 7**).

**Fig 8:**
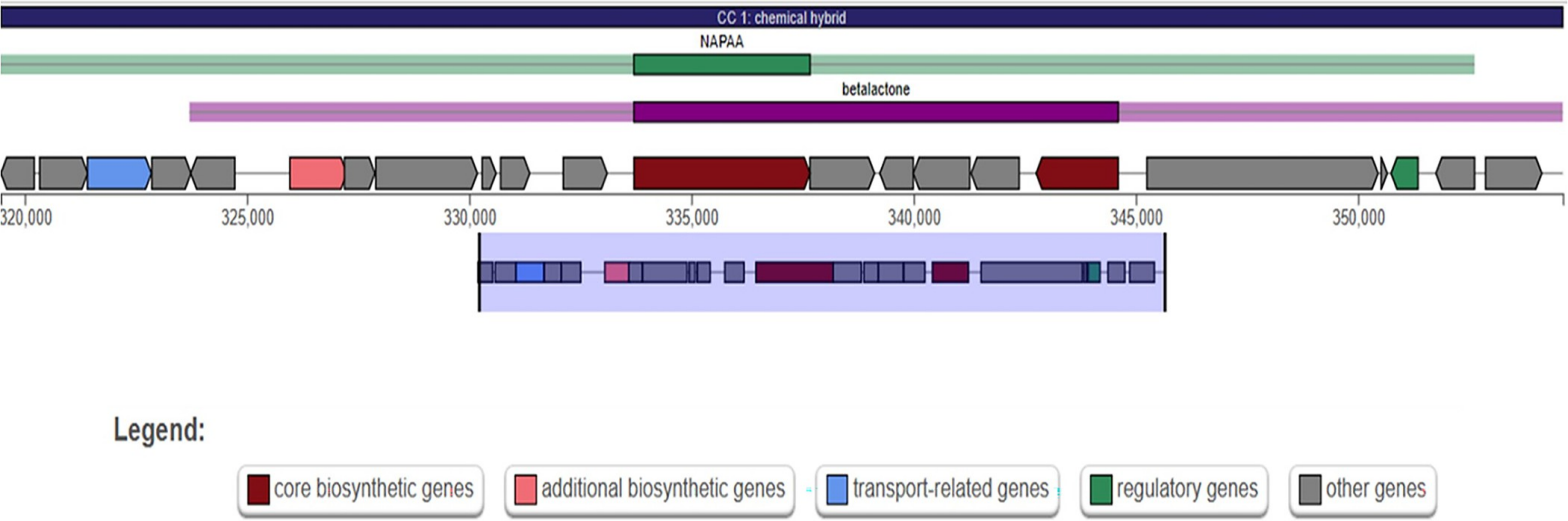
Hybrid BGCs structure *C*. *glutamicum* strains. Hybrid BGCs in four strains harbour same structure of NAPAA-betalactone. The different locations of NAPAA-betalactone are, 256574–294301 base pairs in strain B253, 334064–369207 base pairs in strain R, 319,462–354,607 base pairs in strain SCgG1, and 319,463–354,608 base pairs in SCgG2.

Additionally, strain B253, R, SCgG1, and SCgG2 contain hybrid BGCs. All four strains contained hybrid BGCs comprised with NAPAA and betalactone. But the locations of NAPAA-betalactone hybrid BGCs are different in the genomes. The locations are 256574–294301 base pairs in strain B253, 334064–369207 base pairs in strain R, 319,462–354,607 base pairs in strain SCgG1, and 319,463–354,608 base pairs in SCgG2 (**Fig 8**).

PRISM 4 identified 4 major classes of BGCs which were polyketide, nonribosomal peptide, dehydratase, class II/III confident bacteriocin. Polyketide and nonribosomal peptide BGCs were present in all strains, while dehydratase were found in 21 strains and class II/III confident bacteriocin were found in 12 strains of *C*. *glutamicum* (**S5 Table**).

Besides, genome mining by BAGEL4 revealed bacteriocin coding clusters among 12 strains (**S5 Table**). Our identified BGCs from different online platform for each strain is listed in **Table 4**.

**Table 4:** Different hits of BGCs from different genome mining tools using *C*. *glutamicum* genomes.

Species name	Isolation source	Genome size in Mbp	AntiSMASH Hit	Prism Hit	BAGEL Hit
*C*. *glutamicum* CP	air	3.34	4	4	1
*C*. *glutamicum* XV	soil	3.33	4	4	1
*C*. *glutamicum* AJ1511		3.3	4	4	1
*C*. *glutamicum* ATCC 21799		3.33	4	4	1
*C*. *glutamicum* ZL-6	soil	3.33	4	4	1
*C*. *glutamicum* B253		3.21	6	4	1
*C*. *glutamicum* BE		3.34	5	4	1
*C*. *glutamicum* 14067	rotten onion	3.33	5	4	1
*C*. *glutamicum* YI	soil	3.34	5	4	1
*C*. *glutamicum* R		3.31	5	3	0
*C*. *glutamicum* SCgG1	soil	3.35	5	3	0
*C*. *glutamicum* SCgG2	soil	3.35	5	3	0
*C*. *glutamicum* USDA-ARS-USMARC-56828	mucus of calf	3.25	4	3	0
*C*. *glutamicum* CGMCC1.15647		3.36	4	2	0
*C*. *glutamicum* ATCC 21573		3.18	4	3	0
*C*. *glutamicum* B414	soil	3.16	4	3	0
*C*. *glutamicum* CICC10064	soil	3.16	4	3	0
*C*. *glutamicum* MB001		3.08	4	2	0
*C*. *glutamicum* TQ2223	soil	3.31	4	2	0
*C*. *glutamicum* ATCC 13032		3.37	4	2	0
*C*. *glutamicum* HA		3.35	4	2	0
*C*. *glutamicum* BCA		3.06	3	2	0
*C*. *glutamicum* C1	derivative of ATCC 13032	2.84	2	2	0
*C*. *glutamicum* CR101	lab strain	3.03	4	2	0
*C*. *glutamicum* JH41		3.13	4	2	0
*C*. *glutamicum* AR1		3.15	4	3	0
*C*. *glutamicum* ATCC 21831		3.18	4	3	0
*C*. *glutamicum* TCCC11822	soil	3.3	4	4	1
*C*. *glutamicum* ATCC 13869	soil	3.3	4	4	1
*C*. *glutamicum* WM001	soil	3.32	4	4	1

### 3.5 Genomic and SNP analysis

In this study, we employed BRIG-0.95 for comprehensive genome comparisons among various strains of *C*. *glutamicum*. The reference genome, *C*. *glutamicum* SCgG2, was utilized as a baseline for these comparisons. Notably, a substantial portion of genes present in SCgG2 were found to be shared by the other strains, indicating a core genomic similarity among these strains.

**Fig 9:**
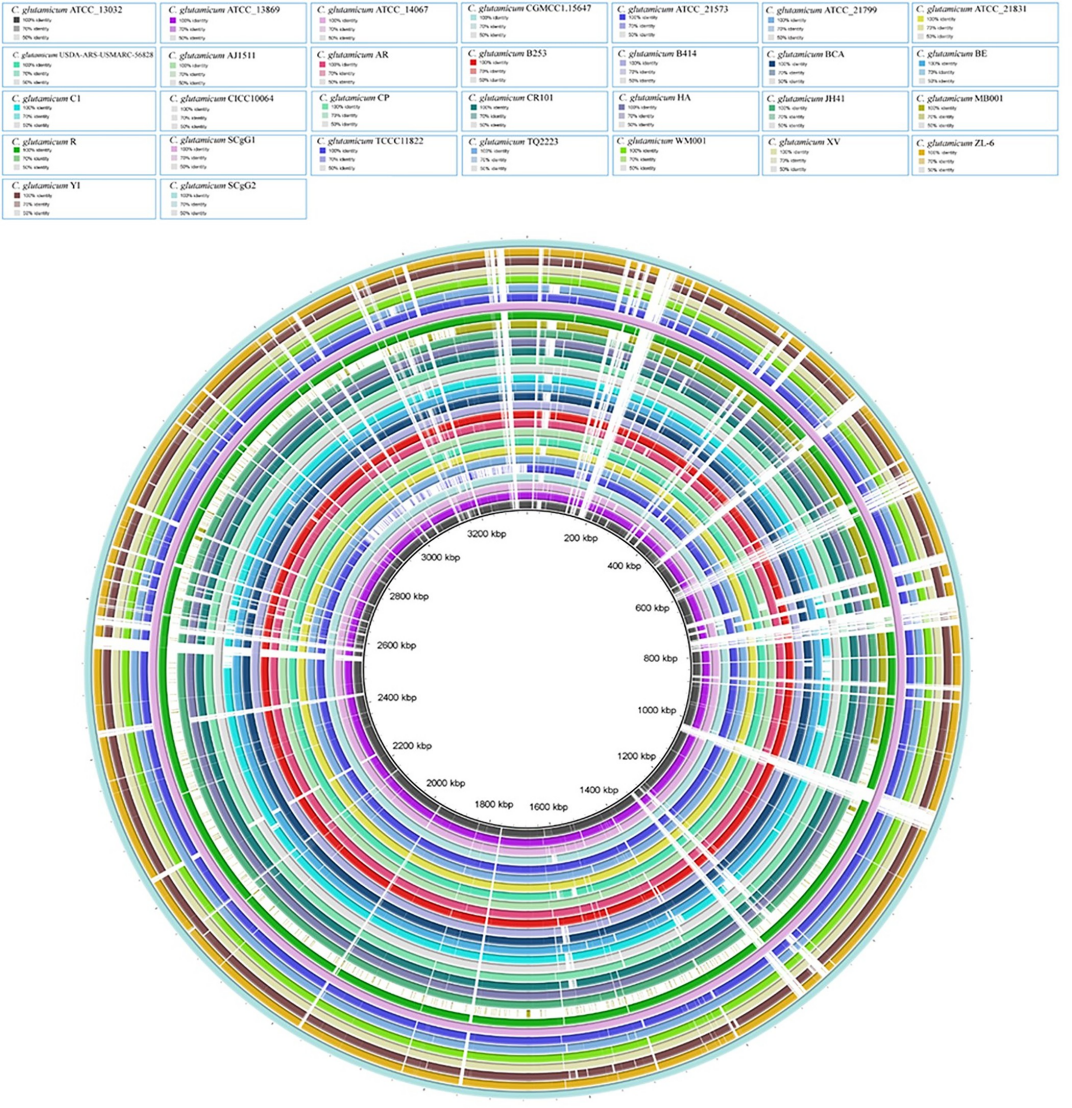
BRIG Diagram illustrating homologous chromosome segments of *C*. *glutamicum* strains using strain SCgG2 as the reference genome.

However, a detailed examination of the genomic alignments revealed significant disparities between SCgG2 and other strains, as denoted by white gaps in Fig 9. These gaps signify regions where genes were absent in certain strains, indicating potential genetic variations. Such discrepancies could be attributed to the integration of mobile genetic elements, horizontal gene transfer events, or recombination phenomena. These mechanisms are known to drive genetic diversification in bacterial populations, leading to the acquisition or loss of specific genes over evolutionary time. The identification of these genomic variances underscores the dynamic nature of *C*. *glutamicum* genomes and highlights the genomic plasticity within this bacterial species.

**Fig 10:**
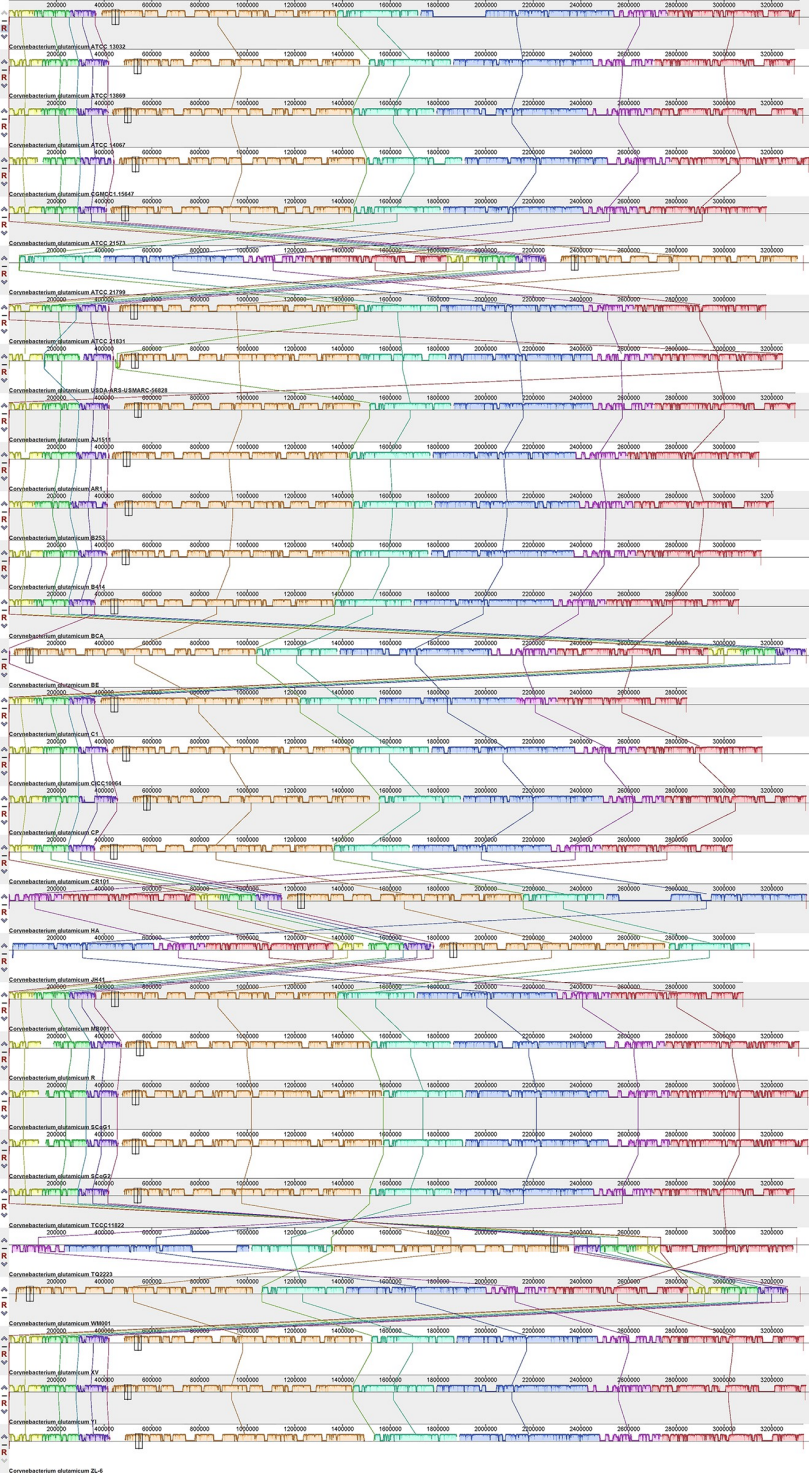
The pairwise whole-genome Mauve alignment analysis revealed substantial structural variations within the circular chromosomes of *C*. *glutamicum* strains.

Fig 10 illustrates the output from pairwise whole-genome Mauve alignments, confirming the presence of significant structural variations among the genomes of the analysed strains. In each comparison, matching coloured blocks and connecting lines delineate homologous genome sections between the compared pairs. Notably, strains TCCC11822, TQ2223, BCA, CR101, HA, and ATCC 21573 exhibited the most significant variations, indicating diverse genomic structures within these strains. These visual cues provide insights into the shared genomic regions and structural differences between the analysed strains.

SNPs analysis within the various *C*. *glutamicum* strains provided valuable insights into the genetic diversity of these strains. **Table 5** presents a comprehensive analysis of genetic variants among various strains of *C*. *glutamicum*. Notably, *C*. *glutamicum* USDA-ARS-USMARC-56828 exhibited the highest number of total variants (41270), characterized by substantial counts in complex variants (7908) and SNPs (32960). This strain displayed a significant divergence compared to others. Conversely, strains like *C*. *glutamicum* SCgG1 showed minimal variants, with only 28 total variants. Several strains, such as *C*. *glutamicum* R, displayed a relatively low total variant count (20433) and a notable prevalence of deletions (141) and insertions (130). These findings underscore the genetic diversity within *C*. *glutamicum* strains, with certain strains exhibiting distinctive patterns of variation, potentially influencing their biological characteristics. The presence of unique SNPs in each strain suggests specific genomic changes, potentially influencing their functional attributes and ecological roles.

**Table 5:** SNPs detected in *C*. *glutamicum* strains.

Strain	Accession Number	Variant					
		Complex	Deletions	Insertions	MNPs	SNPs	Total Variant
***C*. *glutamicum* AJ1511**	AP017557.2	6934	187	206	-	30582	37909
***C*. *glutamicum* ATCC_21799**	AP022856.1	6904	190	204	-	30676	37974
***C*. *glutamicum* SCgG1**	CP004047.1	1	1	-	-	26	28
***C*. *glutamicum* SCgG2**	CP004048.1	Referance					
***C*. *glutamicum* ZL-6**	CP004062.1	7010	187	212	-	31300	38709
***C*. *glutamicum* MB001**	CP005959.1	6938	212	183	-	30316	37649
***C*. *glutamicum* ATCC_21831**	CP007722.1	7032	169	194	-	30780	38175
***C*. *glutamicum* AR1**	CP007724.1	7037	167	195	-	31104	38503
***C*. *glutamicum* B253**	CP010451.1	6646	205	213	-	30181	37245
***C*. *glutamicum* CP**	CP012194.1	6963	188	208	-	30985	38344
***C*. *glutamicum* B414**	CP012297.1	7380	183	188	-	30963	38714
***C*. *glutamicum* CICC10064**	CP012298.1	7389	181	190	-	30983	38743
***C*. *glutamicum* USDA-ARS-USMARC-56828**	CP013991.1	7908	197	205	-	32960	41270
***C*. *glutamicum* YI**	CP014984.1	6704	215	199	-	30639	37757
***C*. *glutamicum* ATCC_13869**	CP016335.1	6982	185	204	4	30676	38051
***C*. *glutamicum* C1**	CP017995.1	6387	200	179	1	28229	34996
***C*. *glutamicum* XV**	CP018175.1	6962	192	209	-	30990	38353
***C*. *glutamicum* TCCC11822**	CP020033.1	6934	185	208	-	30782	38109
***C*. *glutamicum* TQ2223**	CP020658.1	6985	235	183	-	30668	38071
***C*. *glutamicum* ATCC_14067**	CP022614.1	6649	212	201	-	29882	36944
***C*. *glutamicum* ATCC_13032**	CP025533.1	6986	217	186	2	30405	37796
***C*. *glutamicum* HA**	CP025534.1	7019	220	185	2	30376	37802
***C*. *glutamicum* JH41**	CP041729.1	6811	185	193	-	30173	37362
***C*. *glutamicum* BE**	CP053188.1	6640	208	201	-	29779	36828
***C*. *glutamicum* ATCC_21573**	CP068290.1	7170	190	195	-	31222	38777
***C*. *glutamicum* CR101**	CP080542.1	6941	212	193	-	30327	37673
***C*. *glutamicum* R**	NC_009342.1	3520	141	130	-	16642	20433
***C*. *glutamicum* WM001**	NZ_CP022394.1	6991	186	204	-	30871	38252
***C*. *glutamicum* BCA**	NZ_CP059382.1	6934	212	185	-	30146	37477
***C*. *glutamicum* CGMCC1.15647**	NZ_CP073911.1	7366	197	202	-	32021	39786

**Fig 11:**
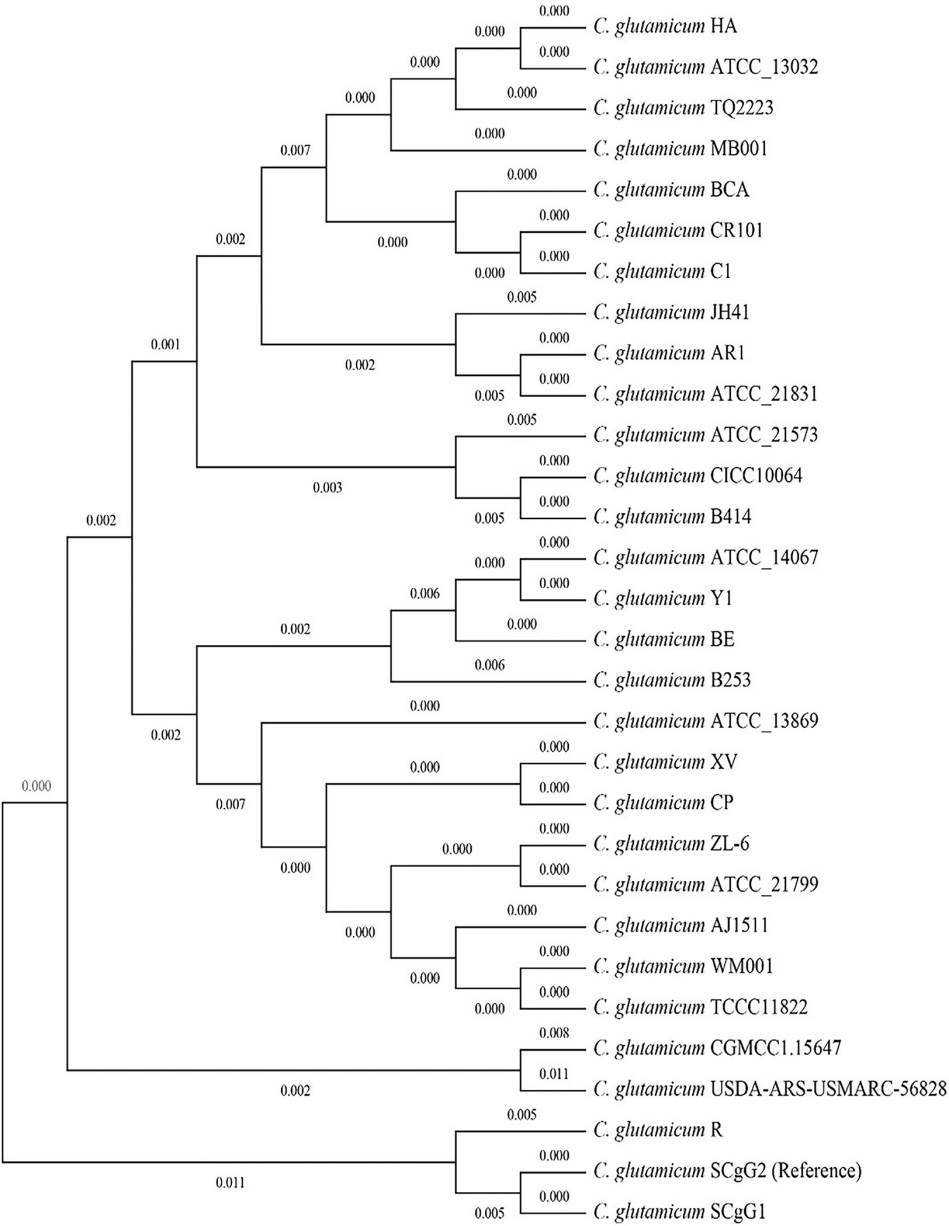
Visualizing the phylogeny of *C*. *glutamicum* strains based on core SNP genes.

The phylogenetic tree, as illustrated in Fig 11 based on Core SNPs analysis, delineates the evolutionary relationships among the *C*. *glutamicum* strains. The tree is rooted with a reference strain (SCgG2). Noteworthy patterns emerge, revealing distinct clusters and branches that denote genetic proximity. For instance, strains like AJ1511, WM001, and TCCC11822 form a cluster, suggesting a shared genetic ancestry. Similarly, ZL-6 and ATCC 21799 exhibit close genetic relatedness. The tree also portrays a bifurcation between B253 and its cluster, including BE, ATCC 14067, and YI, reflecting their divergence. Further branching showcases the genetic relationships among diverse strains, emphasizing the intricate evolutionary dynamics within the *C*. *glutamicum* species. The placement of the reference strain in the analysis enables a comparative understanding of genetic variations, highlighting its pivotal role in contextualizing the evolutionary history of the examined strains. Overall, the phylogenetic tree provides a visual representation of the genetic distances and relationships, offering valuable insights into the evolutionary landscape of *C*. *glutamicum*.

### 3.6 Horizontal gene transfer

**Fig 12:**
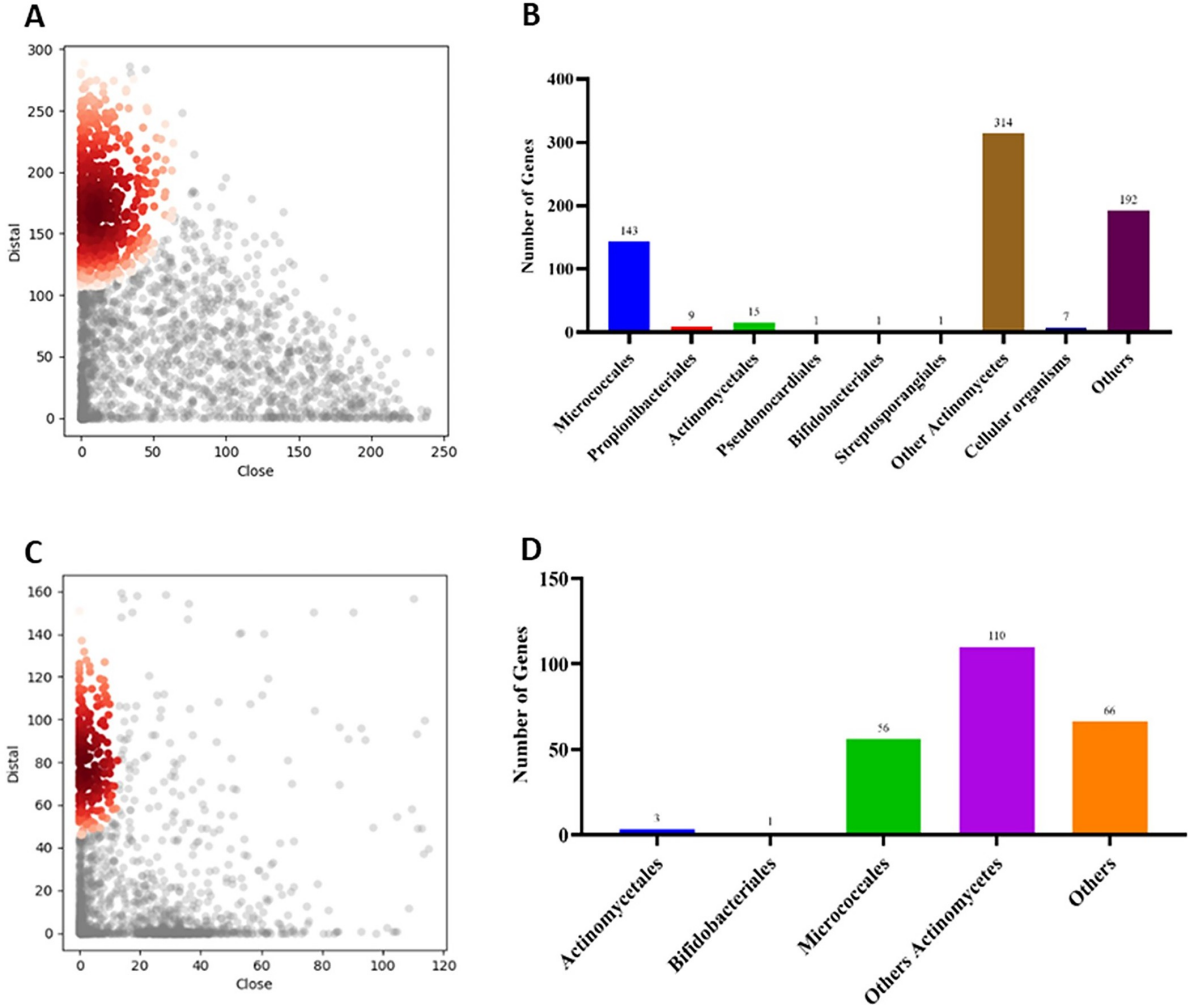
HGT events in *C*. *glutamicum* AJ1511 and AR1 Strains. (A) Scatter plot illustrating horizontally transferred genes in AJ1511 (Colour dots represents horizontally transferred genes and colourless dots represents native genes). (B) Distribution of donor organisms and the corresponding number of genes transferred in AJ1511. (C) Scatter plot showcasing horizontally transferred genes in AR1 (Colour dots represents horizontally transferred genes and colourless dots represents native genes). (D) Distribution of donor organisms and the corresponding number of genes transferred in AR1. (In the scatter plots, coloured dots represent genes transferred through HGT).

Utilizing the HGTector tool, an exhaustive analysis was performed, revealing a substantial number of HGT events within the genomes of *C*. *glutamicum* strains. Specifically, in the AJ1511 strain, 684 distinct HGT events were identified from a dataset of 3014 predicted proteins. These events were predominantly sourced from *Actinomycetes* (71%) and to a lesser extent, *Micrococcales* (21%). Similarly, in the AR1 strain, a total of 237 genes were predicted to have undergone HGT events out of 2759 proteins analysed. Notably, the majority of these events were attributed to *Actinomycetes* (73%), with a smaller fraction originating from *Micrococcales* (23%) as illustrated in Fig 12. Considering the prevalence of HGT events in AJ1511 and AR1, it is likely that other *C*. *glutamicum* strains, would reveal a mosaic of genetic origins. The genomic plasticity observed in these two strains is indicative of the adaptive strategies employed by *C*. *glutamicum* populations, emphasizing the role of HGT in shaping their genetic repertoire.

### 3.7 Pathogenicity, virulence properties and plasmid analysis

The investigation revealed that none of the strains belonging to *C*. *glutamicum* exhibited characteristics indicative of human pathogenicity. A detailed presentation of these findings is encapsulated in the Table 6. This underscores the non-pathogenic nature of the examined *C*. *glutamicum* strains concerning human health. It is noteworthy that non-pathogenic bacteria lack the genetic elements associated with virulence, thereby affirming their incapacity to induce infections or diseases in humans.

**Table 6:** Pathogenicity prediction results for various *C*. *glutamicum* strains.

Starin	Probability of being a human pathogen	Matched Pathogenic Families	Prediction
*C*. *glutamicum* AJ1511	0.258	0	Non-human pathogen
*C*. *glutamicum* ATCC_21799	0.258	0	Non-human pathogen
*C*. *glutamicum* SCgG1	0.265	0	Non-human pathogen
*C*. *glutamicum* SCgG2	0.265	0	Non-human pathogen
*C*. *glutamicum* ZL-6	0.262	0	Non-human pathogen
*C*. *glutamicum* MB001	0.257	0	Non-human pathogen
*C*. *glutamicum* ATCC_21831	0.262	0	Non-human pathogen
*C*. *glutamicum* AR1	0.261	0	Non-human pathogen
*C*. *glutamicum* B253	0.265	0	Non-human pathogen
*C*. *glutamicum* CP	0.257	0	Non-human pathogen
*C*. *glutamicum* B414	0.264	0	Non-human pathogen
*C*. *glutamicum* CICC10064	0.264	0	Non-human pathogen
*C*. *glutamicum* USDA-ARS-USMARC-56828	0.254	0	Non-human pathogen
*C*. *glutamicum* YI	0.261	0	Non-human pathogen
*C*. *glutamicum* ATCC_13869	0.258	0	Non-human pathogen
*C*. *glutamicum* C1	0.256	0	Non-human pathogen
*C*. *glutamicum* XV	0.256	0	Non-human pathogen
*C*. *glutamicum* TCCC11822	0.257	0	Non-human pathogen
*C*. *glutamicum* TQ2223	0.255	0	Non-human pathogen
*C*. *glutamicum* ATCC_14067	0.261	0	Non-human pathogen
*C*. *glutamicum* ATCC_13032	0.256	0	Non-human pathogen
*C*. *glutamicum* HA	0.257	0	Non-human pathogen
*C*. *glutamicum* JH41	0.26	0	Non-human pathogen
*C*. *glutamicum* BE	0.261	0	Non-human pathogen
*C*. *glutamicum* ATCC_21573	0.266	0	Non-human pathogen
*C*. *glutamicum* CR101	0.256	0	Non-human pathogen
*C*. *glutamicum* R	0.26	0	Non-human pathogen
*C*. *glutamicum* WM001	0.258	0	Non-human pathogen
*C*. *glutamicum* BCA	0.256	0	Non-human pathogen
*C*. *glutamicum* CGMCC1.15647	0.259	0	Non-human pathogen

The plasmid analysis across different strains of *C*. *glutamicum* revealed diverse characteristics. Strains CP, XV, B253, USDA-ARS-USMARC-56828, AR1, ATCC_21831, and ATCC_13869 were found to harbor IncA/C type plasmids, with varying lengths and GC content (Table 7). Notably, strains R and CGMCC1.15647 exhibited distinct plasmid types, namely IncI1 and IncHI1, respectively, and displayed substantial variations in plasmid sizes. The gene content of these plasmids varied among strains, encompassing differences in coding sequences (CDs), pseudo genes, CRISPR arrays, rRNAs, tRNAs, ncRNA, and frameshifted genes. Among the strains analyzed, 10 were reported to carry single plasmids, while C. glutamicum CGMCC1.15647 was unique with two plasmids. The prevalent IncA/C type plasmid, found in the majority of strains, is known for its role in modulating changes to bacterial host chromosomes. In contrast, *C*. *glutamicum* R carries an IncI1 type plasmid, responsible for encoding sex pili in bacteria. IncHI1 type plasmid is associated with antibiotic resistance. This comprehensive analysis underscores the diversity and functional significance of plasmids in *C*. *glutamicum* strains.

**Table 7:** Plasmid characteristics in different *C*. *glutamicum* strains.

Strain	Plasmid Accession Number	Type of Plasmid	Length	GC (%)	Genes	CDs	Pseudo Genes	CRISPR Arrays	rRNAs	tRNAs	ncRNA	Frameshifted Genes
*C*. *glutamicum* CP	CP012412.2	IncA/C	4447	53.5	3122	2898	145		6	60	1	62
*C*. *glutamicum* XV	CP018174.1	IncA/C	4447	53	3165	3079	193		6	65	3	
*C*. *glutamicum* B253	CP010452.1	-	2175	51	2984	2767	138	3	18	60	1	54
*C*. *glutamicum* R	AP009045.1	IncI1	49120	53.9								
*C*. *glutamicum* USDA-ARS-USMARC-56828	CP014329.1	IncA/C	48334	53.5	3062	2981	85	1	6	60	3	
*C*. *glutamicum* CGMCC1.15647	CP073912.1		47523	58								
	CP073913.1	IncHI1	24235	54								
*C*. *glutamicum* AR1	CP007725.1	IncA/C	16810	51.5	2901	2760	65		18	57	1	36
*C*. *glutamicum* ATCC_21831	CP007723.1	IncA/C	16810	51.5	2932	2785	68		18	60	1	42
*C*. *glutamicum* ATCC_13869	CP016336.1	IncA/C	4447	53.5	3075	2994	108		6	60	3	

## 4. Discussion

Whole genome comparison by ANI calculation revealed high degree of relatedness between *C*. *glutamicum* strains. ANI computation with a higher than 97% score verifies that our studied genomes belong to the same species and are closely related. ANI comparison between *Corynebacterium cystitidis* strains showed a 95.1% score when isolated from the different hosts but showed a >99% score when isolated from the same host [65]. Our demographic data also support a >97% score since most of our strains are from soil sources.

The average genome size (3.24 Mbp) of the strains was slightly high, compared with non-pathogenic *C*. *casei* LMG S-19264 (3.11 Mbp) and *C*. *efficiens* YS-314 (3.15 Mbp) [66]. Moreover, the average number of genes (3197) was also higher than *C*. *casei* LMG S-19264 (2872) and *C*. *efficiens* YS-314 (3064) [66]. On the other hand, the average GC content was lower among *C*. *glutamicum* strains (54.15%) than other non-pathogenic *C*. *variabile* DSM 44702 (76.1%) and *C*. *efficiens* YS-314 (69.93%) [67]. We found variation in tRNA coding genes among the *C*. *glutamicum* strains, since the tRNA genes varied from 57 to 65 among the strains. Whereas, *C*. *variabile* DSM 44702 and *C*. *efficiens* YS-314 contains 59 and 56 tRNA genes respectively [67]. Additionally, *C*. *glutamicum* strains possess more rRNA genes (15–18 rRNA genes) compared with other non-pathogenic *Brevibacterium auranticum* strains and *Brevibacterium linens* ATCC 19391 (12 rRNA genes) [68]. Besides, the average CDS among the strains was 3007, comparatively higher than *C*. *efficiens* YS-314 (2950 CDS) [69].

Pan-genome study of *Corynebacterium* at genus level showed very low number of core genes [66,67]. Analysis between 51 strains of various pathogenic and non-pathogenic species of *Corynebacterium* genus showed 8.69% of core genes [66]. Similarly, study of eleven *Corynebacterium* species showed 6.68% of core genes [67]. Contrary to genus level, we found core genes of 29.1% at sub-species level among *C*. *glutamicum* strains, which is somewhat higher than *C*. *pseudotuberculosis* core genes (26.1%) at sub-species level [70]. The number of cloud genes (strain-specific genes) was considerably large and covered 47.78% of the pan-genome, similar to *C*. *pseudotuberculosis* cloud genes (42.34%) [70]. The low percentage of core genes in *C*. *glutamicum* species likely results from a combination of factors such as horizontal gene transfer, adaptation to diverse environments, evolutionary divergence, and specialization. From an evolutionary perspective, this genetic diversity contributes to the species’ ability to adapt, survive, and thrive in different ecological niches. Which strongly demonstrates the diversity among the strains. Large accessory genomes and a high number of strain-specific genes are frequently linked to horizontal gene transfer (HGT) in microorganisms [71]. Besides, we found low GC content of *C*. *glutamicum* strains with other non-pathogenic species of *Corynebacterium* genus. Our study also suggests a clear inverse relation between the abundance of accessory genes and the genomic GC content. Specifically, as the GC percentage increases, there is a notable decrease in the number of accessory genes observed. This finding supports the idea of possible relation of low GC content with horizontal gene transfer and codon reassignment of *C*. *glutamicum* [72–75].

Our study shows the open nature of the *C*. *glutamicum* pan-genome, which indicates that new gene families continuously will be added to the pan-genome. The open pan-genome of *Corynebacterium* at genus level was also reported by the pan-genomic analysis of 40 strains of eleven different *Corynebacterium* species [76]. Thus, the pan-genome of *C*. *glutamicum* indicates the diversity of the gene pool and the likeliness of increasing gene number.

Another objective of our study was to uncover the diversity and distribution of BGCs among the strains. Although BGCs producing metabolic products remained undocumented, predictions based on bioinformatics revealed that several of them might encode products with unique structures [77–79]. Thus, our computational approaches were to predict BGCs as a screening process for new bioactive compound production, which are to be effectively applied in the wet laboratories.

NAPAA of Nonribosomal peptide synthetases (NRPs) gene clusters and T1PKS of Polyketide synthases (PKSs) gene clusters were found in all the studied strains. Additionally, Terpene BGCs were found in 96.67% strains. T1PKS, Terpene, NAPAA and other NRPs were also most common in *Gordonia hongkongensis* EUFUS-Z298 [80], *Burkholderia* spp. [18], in activated sludge microbiome [81], and in *Ktedonobacteria* [82]. NAPAA, particularly e-poly-lysine, demonstrate notable antimicrobial efficacy, showcasing widespread utility in the food and pharmaceutical sectors. Conversely, T1PKS harbor the capability to biosynthesize peptides with antibiotic and antitumor properties. Terpenoids exhibit robust and specific biological activities, notably against diseases such as cancer and malaria. The consistency of limited number of BGCs among closely related bacterial population was previously reported [83]. Which indicates that BGCs ‘fixation’ can be occurred as a strong positive selection and to survive specific environment by the activity of encoded products [17]. The novel BCGs identified from the strains used for analysis include betalactone and lanthipeptide class IV BGCs. Betalactone BGCs were predicted in strain R, B253, SCgG1, and SCgG2, while lanthipeptide class IV BGCs were only found in strain B253. Betalactones manifest noteworthy bioactivity against bacteria, fungi, and cancer cell lines. Lanthipeptides, belonging to the subclass of ribosomally-synthesized and posttranslationally-modified peptides (RiPPs), generally display feeble antibacterial activities, with Lenthipeptide-class-IV standing out as a noteworthy example. A study of *Bacillus cereus* strains identified different lanthipeptide classes, and concluded that several lanthipeptide classes can evolve independently, and most of the lanthipeptide BGCs can originated from intra-species horizontal gene transfer [84].

Additionally, PKS and NRPs BGCs which were most common in our studied genomes, are considered as representatives of two major classes of antibiotics [80]. Kalimantacin antibiotics with strong antistaphylococcal effect, from *Alcaligenes* species YL-02632S [85,86] and antibiotic batumin from *Pseudomonas batumici* have been produced utilizing these BGCs [87]. *C*. *glutamicum* is suitable for T1PKS and NRPs synthesis by heterologous expression since it possesses endogenous 4’-phosphopantetheinyl transferase (PPTase), PptAcg [88]. Roseoflavin, a broad-spectrum antibiotic was already produced using *C*. *glutamicum* via the heterologous expression of its BGCs [89]. We also found bacteriocin gene clusters in 12 strains of *C*. *glutamicum*. Bacteriocins have been seen as a feasible alternative to traditional antibiotics because of their distinct antibacterial processes. Besides, it can be used as innovative carrier molecules [90] and also as plant growth-promoting agent, antiviral agent, and anti-cancer agents [91].

Whole genome comparison based on ANI scores also revealed the phylogenetic relationship among the strains. We divided all 30 strains into five clades. Clade 1 with five strains, clade 2 with seven strains, clade 3 with eight strains, clade 4 with four strains, and clade 5 with six strains. We have seen diversity of the BGCs among clade 1, clade 2, and clade 4. Whereas members of clade 3 and clade 5 contain the same number of BGCs, but these two clades harbour different BGCs. We observed similar BGCs among the soil isolated strain CICC10064, B414, and TQ2223. Similarly, soil isolated strain XV, ZL-6, YI, TCCC11822, ATCC 13869, and WM001 have similar BGCs, where strain YI have gained extra NAPAA class. Soil isolated strain SCgG1 and ScgG2 have similar BGCs class with betalactone. On the other hand, strain C1, which is an engineered derivative of ATCC 13032 have lost double Terpene BGCs.

Additionally, we identified NAPAA-betalactone hybrid BGCs among strain B253, R, SCgG1 and SCgG2. Hybrid BGCs encodes genes that are responsible for multiple scaffold-synthesizing enzymes [92,93]. Occurrence of hybrid BGCs are common for some bacteria (98% occurrence in *Streptomyces*) [94], yet the exact roles of hybrid BGCs are not completely known [95,96]. It is noteworthy, that the specific locations of these hybrid BGCs within the genomes of these strains exhibit variation, as illustrated in Fig 8. This disparity implies that these hybrid BGCs might have undergone acquisition or rearrangement through horizontal gene transfer or recombination events, thereby contributing to genomic diversity across the strains. Consequently, our assertion of identifying hybrid BGCs is rooted in their gene content and functional characteristics, rather than their precise physical placement within the genomes.

We found that the number of BGCs is positively correlated with the genome size and the gene number of the strains. Strain SCgG1, ScgG2, BE, YI, 14067, and strain R with larger genome size and with high number of genes, each harbouring 5 BGCs in their genomes. Though, strain CGMCC1.15647 with the highest gene number and the largest genome size contains 4 BGCs. Thus, our correlation regression analysis shows that if the genome size and the gene number increase, the number of BGCs is more likely to increase. Generally, strains with larger genomes tend to exhibit a higher number of BGCs, a phenomenon attributed to the potential accumulation of accessory genes and genomic islands carrying BGCs [97].

The potential presence of sequencing errors within publicly available databases remains a notable concern. Only complete genome sequences of *C*. *glutamicum* strains, which enhance the reliability and comprehensiveness of the study’s findings, were considered, addressing the potential presence of sequencing errors within publicly available databases. Prokka and FragGeneScan were employed for genome annotation and gene prediction, representing widely used and validated tools for prokaryotic genomes. To ensure robust genome comparison and species delineation, OrthoANI, a pairwise average nucleotide identity (ANI) algorithm, more robust and accurate than traditional methods, was utilized. The pan-genome analysis employed Roary, BPGA, and USEARCH, utilizing rigorous computational frameworks and sequence identity cut-offs for gene classification and estimation. This comprehensive approach aimed to mitigate concerns related to sequencing errors, enhance reliability, and employ validated tools for effective genome annotation and pan-genome analysis in the study of *C*. *glutamicum* strains. Nevertheless, our investigation has revealed discernible diversity across various genomic features among the strains, along with variations in the abundance of biosynthetic gene clusters (BGCs) within their genomes. Virulence genes are pivotal elements that contribute to the pathogenicity of microorganisms, enabling them to induce diseases. In contrast, BGCs are typically responsible for encoding enzymes and proteins involved in synthesizing specific secondary metabolites, such as T1PKS, Terpene, NAPAA, betalactone, and lanthipeptide. The connection between BGCs and virulence is diverse, as certain secondary metabolites produced by BGCs can influence the virulence of microorganisms.

However, in our investigation, no identifiable secondary metabolites produced by BGCs were associated with virulence. Remarkably, all examined strains were found to be non-pathogenic. This suggests that there might be an absence of virulence genes located within the BGCs of these strains. The collective non-pathogenic nature of the strains reinforces the notion that the BGCs under scrutiny may not harbor genes contributing to virulence, further emphasizing the safety profile of these microorganisms in the context of human health.

While our study successfully identified numerous distinct polymorphic sites among the strains under investigation, it is crucial to acknowledge a limitation. The specific interaction or overlap between these polymorphic sites and BGCs in *C*. *glutamicum* has not been thoroughly explored within the scope of our research. This unexplored aspect represents a noteworthy limitation, suggesting a promising avenue for future investigation.

In all, we can say that strains of *C*. *glutamicum* can be a good candidate for engineering to produce various novel compound through BGCs expression. Also the strain may have potential to produce antibiotic, plant growth promoting agent, antiviral agent and anti-cancer agent.

## 5. Conclusions

Our objectives of the study were to elucidate the genetic variation, pan-genomic characteristics, and distribution of BGCs among 30 strains of *C*. *glutamicum*. We observed genetic variation and diversity in the BGCs distribution. Pan-genomic study of *C*. *glutamicum* strains revealed diversity at the sub-species level. We found a large number of strain-specific genes and the open nature of the *C*. *glutamicum* pan-genome. This study has yielded valuable insights into previously unexplored biosynthetic gene clusters (BGCs) that play a role in the production of betalactones, lanthipeptides, and NAPAA-betalactone hybrids. Thus, we conclude that various strains of *C*. *glutamicum* should be on focus for the discovery of natural drugs at the industrial level.

## Supporting information

S1 Table Average Nucleotide Identity (ANI) values for *C*. *glutamicum* strains. (XLSX)

S2 Table Genomic characteristics of various *C*. *glutamicum* strains. (XLSX)

S3 Table Biosynthetic Gene Clusters (BGCs) and corresponding hit counts. (XLSX)

S4 Table BGCs distribution across *C*. *glutamicum* strains. (XLSX)

S5 Table BGCs and genomic characteristics comparison of various *C*. *glutamicum* strains. (XLSX)
